# Exploring the interaction between sport type and competitive status in cognitive performance

**DOI:** 10.3389/fpsyg.2026.1915999

**Published:** 2026-08-17

**Authors:** Juraj Krkošek

**Affiliations:** Department of Physical Education and Social Sciences, Faculty of Sports Studies, Masaryk University, Brno, Czechia

**Keywords:** closed-skill sports, cognitive performance, impulsivity, open-skill sports, reaction time, Stroop interference

## Abstract

**Background:**

Cognitive performance, including reaction time, inhibitory control, and impulsivity, represents an important component of both athletic and everyday functioning. However, the relationship between different types of sports participation and competitive engagement remains insufficiently understood.

**Objective:**

The present study aimed to examine the effects of sport type (open vs. closed skill) and competitive status (competitive vs. non-competitive) on cognitive performance.

**Methods:**

A total of 30 participants, including university students and amateur athletes, completed a battery of assessments measuring reaction time, interference control using the Stroop test, and impulsivity. Participants were divided according to sport type and competitive involvement. A series of two-way analyses of variance (ANOVA) was conducted to evaluate the main and interaction effects of sport type and competitive status on the measured variables.

**Results:**

No statistically significant main effects of sport type or competitive status were identified across the assessed cognitive variables. The interaction between sport type and competitive status was not statistically significant for reaction time or any other measured outcome.

**Conclusion:**

No significant differences in cognitive performance were observed across sport type or competitive status. Future studies with larger and more balanced samples are needed to further investigate the relationships between sport participation characteristics and cognitive outcomes.

## 1 Introduction

A current trend in sport and exercise psychology research is the increasing interest in the cognitive and psychological differences associated with participation in open- and closed-skill sports. Existing evidence suggests that sports performed in dynamic, externally paced, and unpredictable environments may stimulate cognitive functions more substantially than sports performed under relatively stable and predictable conditions. In particular, open-skill sports appear to place greater demands on executive functions such as inhibitory control, decision-making, attentional processes, and cognitive flexibility due to the constant need to adapt to rapidly changing environmental stimuli (Pačesová, 2021). Nevertheless, it remains unclear to what extent these differences are attributable to the inherent characteristics of the sport itself or to additional factors such as training volume, competitive experience, sport expertise, and individual athlete predispositions. Although previous studies have examined cognitive differences between competition levels and between participants in open- and closed-skill sports, considerably less is known about the combined influence of sport type (open or closed skill) and competitive status on cognitive performance. Moreover, much of the existing evidence has been obtained from competitive athletes, including elite athletes, whereas less attention has been given to non-competitive populations, such as recreational athletes.

One way to categorize sport types is as open- or closed-skill activities; many disciplines exhibit characteristics of both. Consequently, the distinction between open and closed skills should not be viewed as a strict dichotomy, but rather as a continuum, with numerous sports occupying intermediate positions depending on task constraints, environmental variability, and situational demands (Heilmann et al., 2022). For example, even predominantly closed-skill sports may include open-skill components under competitive conditions, while open-skill sports often involve repetitive, highly automated movement patterns.

Executive functions (EFs) are a broad set of higher-order cognitive processes that enable individuals to regulate goal-directed behavior, adapt to novel situations, and override automatic or routine responses. These functions are commonly divided into “Cool EF” and “Hot EF,” reflecting distinct aspects of self-regulation. Cool executive functions are primarily associated with rational, decontextualized, and cognitively demanding processes such as working memory, attentional control, and problem-solving. In contrast, Hot executive functions involve emotional, motivational, and reward-related processes that become particularly relevant in situations characterized by uncertainty, stress, risk, or competitive pressure (Zhou et al., 2012).

Previous research indicates that elite and competitive athletes generally demonstrate superior performance across several domains of executive functioning, particularly in tasks involving attentional control, dual-task processing, rapid decision-making, inhibitory control, working memory, and cognitive flexibility (Ren et al., 2024). Hagyard et al. (2021) conclude that inhibitory control is directly associated with sport performance, increases with higher levels of athletic expertise, and develops progressively over time. Prolonged engagement in sports may facilitate improvements in inhibitory control, which, in turn, may enhance sports performance. However, the existing findings are not entirely consistent across all executive function measures or across different types of sports. Open-skilled athletes show broader advantages across executive function domains, whereas closed-skilled athletes tend to exhibit stronger inhibitory control specifically (Ren et al., 2024; Scharfen and Memmert, 2019). Holfelder et al. (2020) emphasize that cognitive functioning in sport is a complex, multidimensional construct and suggest that currently used assessment tools may not be sufficiently sensitive to fully capture differences among athletes of varying expertise levels, especially with respect to Hot EF processes and impulsivity.

The importance of physical activity, regardless of competitive level, is strongly reflected in cognitive functioning more broadly. Regular participation in sport and physical activity has been associated with improvements in attention, working memory, inhibitory control, decision-making, and other executive functions, as well as more efficient functioning of the prefrontal cortex, a brain region critically involved in behavioral regulation, planning, self-control, and problem-solving (Mandolesi et al., 2018). These positive effects have been observed not only in competitive and elite athletes, but also in non-competitive athletes, including recreational athletes and exercisers, with regular physical activity linked to enhanced cognitive performance and greater mental flexibility. Evidence from Ludyga et al. (2020) suggests that long-term exercise produces a small but positive overall effect on cognition, with benefits appearing to be general rather than domain-specific. Importantly, the magnitude of these effects may depend on moderating factors such as sex, exercise type, and exercise dosage. Coordinative forms of exercise appear to yield greater cognitive benefits, while longer interventions combined with longer session durations are associated with stronger effects, suggesting that the cognitive outcomes of physical activity may be optimized through appropriate exercise selection, intensity, duration, and progression over time (Ludyga et al., 2020).

The cognitive demands associated with open-skill sports may be particularly pronounced among competitive athletes, who are repeatedly exposed to high-pressure situations requiring rapid decision-making, anticipation, and inhibitory control. Therefore, the relationship between open or closed skill sports and cognitive performance may be moderated by competitive status.

## 2 Methods

### 2.1 Participants

A total of 30 participants aged 19–41 years (M = 23.97, SD = 6.21) were included in the study. The sample consisted of 18 males and 12 females. Participants were recruited using convenience sampling from students of Masaryk University in Brno and members of a local football club competing in the 7th division.

Participants were included if they engaged in sports or physical activities in their leisure time or in a training session at least once per week, as self-reported in the physical activity questionnaire (IPAQ). Participants were classified into open-skill (*n* = 11) and closed-skill sport (*n* = 19) groups. Sport type, or physical activity, classification (open vs. closed skill) was determined based on each sport’s predominant characteristics. Open-skill sports were defined as those performed in dynamic, unpredictable environments, whereas closed-skill sports were characterized by stable, predictable conditions (Heilmann et al., 2022). When participants reported involvement in multiple sports or physical activities, classification was determined by the sport that accounted for the greatest proportion of their weekly activity frequency. Thus, the activity type assignment reflected the participant’s predominant exposure to either open-skill or closed-skill activities. The classification of the physical activities and sports included in our research is shown in Table 1.

**Table 1:** Sports and physical activities represented in the sample and their classification according to the open-skill/closed-skill framework.

Sport	*n*	Classification	Reasoning
Weightlifting	8	Closed-skill	Performed in a stable and predictable environment with self-paced and pre-planned movements.
Jogging	5	Closed-skill	Conducted under relatively predictable environmental conditions with repetitive movement patterns.
Cycling	4	Closed-skill	Performance relies primarily on self-regulated and repetitive motor execution in a predictable environment.
Dancing	1	Closed-skill	Movements are generally pre-planned and performed in a structured environment with limited unpredictable external stimuli.
Hiking	1	Closed-skill	Activity is predominantly self-paced and does not require continuous adaptation to rapidly changing external stimuli.
Football	6	Open-skill	Requires continuous adaptation to teammates, opponents, and a dynamically changing environment.
Volleyball	2	Open-skill	Performance depends on reacting to unpredictable game situations and opponent actions.
Baseball	1	Open-skill	Requires anticipation, decision-making, and responses to changing external stimuli.
Floorball	1	Open-skill	Involves rapid perception–action coupling and continuous adaptation to game situations.
Basketball	1	Open-skill	Requires ongoing decision-making and adaptation in a dynamic, unpredictable environment.

The classification reflects the predominant characteristics of each sport or physical activity, acknowledging that open- and closed-skill activities are generally considered to exist along a continuum rather than as strictly discrete categories.

Additionally, participants were categorized as competitive (*n* = 17) or non-competitive (*n* = 13) using criteria adapted from McKinney et al. (2019). While the original framework incorporates both competitive participation and weekly training volume, the present study prioritized competitive involvement as the primary criterion for determining competitive status. Accordingly, participants were classified as competitive if they were registered members of a sports club, regularly participated in official competitions, and reported involvement in at least one sport-specific training session per week. Individuals who did not engage in recreational physical activity (*n* = 2) were retained in the sample due to their exclusive participation in activities related to their registered athlete status (e.g., training sessions). The final representation of participants is shown in Table 2.

**Table 2:** Participant characteristics by sport type and competitive status.

Group	*n*	M/F	Age (M ± SD)
Open-skill	11	8/3	27.45 ± 7.13
Closed-skill	19	10/9	21.95 ± 4.70
Competitive	17	14/3	26.53 ± 7.26
Non-competitive	13	4/9	20.62 ± 1.12
Open-skill competitive	8	7/1	25.38 ± 5.18
Closed-skill competitive	9	7/2	27.56 ± 8.92
Open-skill non-competitive	3	1/2	20.00 ± 0.00
Closed-skill non-competitive	10	3/7	20.80 ± 1.23

M, mean; SD, standard deviation; M/F, male/female.

The study was approved by the Ethics Committee of Masaryk University. All participants provided informed consent prior to participation.

### 2.2 Measures

The testing protocol comprised a personality assessment (Big Five Inventory – IPIP), a self-report physical activity questionnaire (International Physical Activity Questionnaire – Long Form), a cognitive function test (Stroop test, Czech paper version provided by Hogrefe – Testcentrum, Prague), and a reaction time assessment using the BlazePod® system.

Each participant completed one trial of the Stroop test. Interference score (IF) was calculated using the standard Stroop paradigm. The observed performance in the incongruent condition (color–word task; BS), in which participants were required to read color words printed in incongruent ink colors (e.g., the word “GREEN” printed in red), was corrected for baseline performance. The expected score (BS′) was computed as (S × B) / (S + B), where S represents performance in the word-reading condition and B represents performance in the color-naming condition. The interference score was then calculated as IF = BS − BS′.

**Figure 1:**
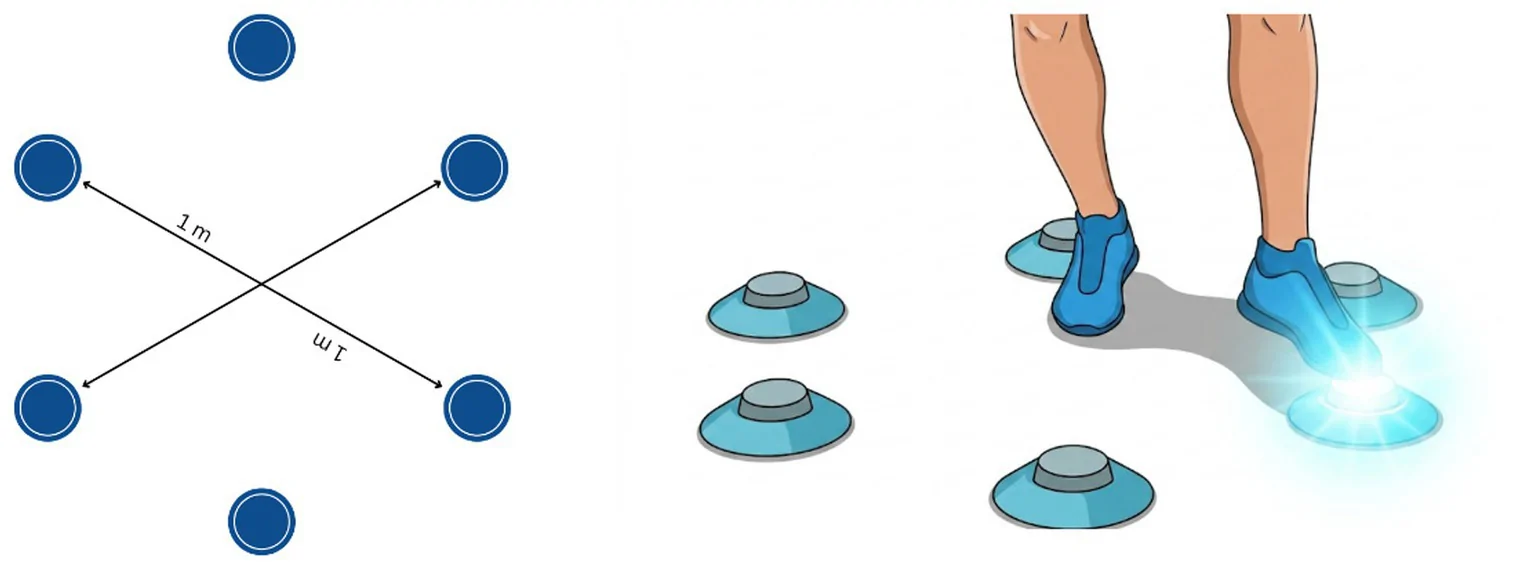
BlazePod test arrangement based on the FIFA 360° Challenge protocol.

Reaction time was assessed using the BlazePod light-based system. The pod arrangement followed the FIFA 360° Challenge protocol and was configured in a diamond formation (Figure 1). Due to the limited procedural details available in the original protocol description, the assessment was implemented according to the demonstrated task execution and pod configuration. During a 30-s trial, visual stimuli were presented sequentially (one at a time), and participants responded by touching the illuminated pod using their feet. Participants maintained their initial orientation throughout the trial and were not permitted to turn toward stimuli presented behind them. The BlazePod system recorded successful contacts and automatically calculated the corresponding mean reaction time (ms), which was used as the dependent variable in all statistical analyses. Two trials were completed, and the better performance (i.e., lower mean reaction time) was retained for analysis.

### 2.3 Procedures

Participants from Masaryk University were assessed in a university gymnasium, whereas football players were tested at their respective club facilities. All assessments were administered individually under standardized conditions. The cognitive function test was conducted in a separate room to ensure a controlled, distraction-free environment.

### 2.4 Statistical analyses

Statistical analyses were conducted using IBM SPSS Statistics (version 31). The level of statistical significance was set at *α* = 0.05.

Descriptive statistics (means and standard deviations) were calculated for all variables. Prior to inferential analyses, the data were screened for outliers and tested for normality using the Shapiro–Wilk test and visual inspection of Q–Q plots. Homogeneity of variances was assessed using Levene’s test.

To examine the effects of sport type and competitive status, a two-way analysis of variance (ANOVA) was conducted with sport type (open-skill vs. closed-skill) and competitive status (competitive vs. non-competitive) as between-subjects factors. Separate analyses were performed for each dependent variable: reaction time performance, Stroop interference score, and impulsivity.

Effect sizes were reported as partial eta squared (ηp^2^). All tests were two-tailed. Given the relatively small and unequal group sizes, effect sizes were interpreted alongside *p*-values.

## 3 Results

Descriptive statistics for interference score, reaction time, and impulsivity across sports types and competitive status groups are presented in Table 3.

**Table 3:** Descriptive statistics for cognitive outcomes by sport type and competitive status.

Group	*n*	Interference score (M ± SD)	Reaction time (ms) (M ± SD)	Impulsivity (M ± SD)
Open-skill competitive	8	9.58 ± 8.40	611.50 ± 37.03	5.63 ± 3.54
Open-skill non-competitive	3	11.83 ± 10.77	672.67 ± 79.19	9.33 ± 6.35
Closed-skill competitive	9	9.35 ± 7.67	704.56 ± 126.91	6.22 ± 3.35
Closed-skill non-competitive	10	14.98 ± 7.35	630.30 ± 67.36	5.50 ± 4.63

Values are presented as mean ± standard deviation (M ± SD). Reaction time is reported in milliseconds (ms).

A series of two-way analyses of variance (ANOVA) was conducted to examine the effects of sport type (open vs. closed) and competitive status (competitive vs. non-competitive) on interference score, reaction time, and impulsivity. Levene’s tests indicated that the assumption of homogeneity of variances was met for all dependent variables (all *p* > 0.05). A summary of the ANOVA results is presented in Table 4.

**Table 4:** Results of two-way ANOVA examining the effects of sport type and competitive status on cognitive and behavioral variables.

Variable	Effect	*F*(₁,₂₆)	*p*	ηp^2^
Interference score (IF)	Sport type	0.20	0.662	0.007
	Competitive status	1.43	0.242	0.052
	Interaction	0.26	0.613	0.010
Reaction time (ms)	Sport type	0.52	0.477	0.020
	Competitive status	0.04	0.854	0.001
	Interaction	3.71	0.065	0.125
Impulsivity	Sport type	0.90	0.351	0.034
	Competitive status	0.77	0.389	0.029
	Interaction	1.69	0.205	0.061

F, F-statistic; p, probability value; ηp^2^ = effect size (partial eta squared).

Results indicated that non-competitive participants had higher interference scores than competitive participants. Similarly, participants engaged in closed-skill sports demonstrated slightly higher interference scores than those in open-skill sports. However, there was no significant main effect of sport type, *F*₍₁,₂₆₎ = 0.20, *p* = 0.662, η_p_^2^ = 0.007, nor of competitive status, *F*₍₁,₂₆₎ = 1.43, *p* = 0.242, η_p_^2^ = 0.052. The interaction between sport type and competitive status was also not significant, *F*₍₁,₂₆₎ = 0.26, *p* = 0.613, η_p_^2^ = 0.010.

**Figure 2:**
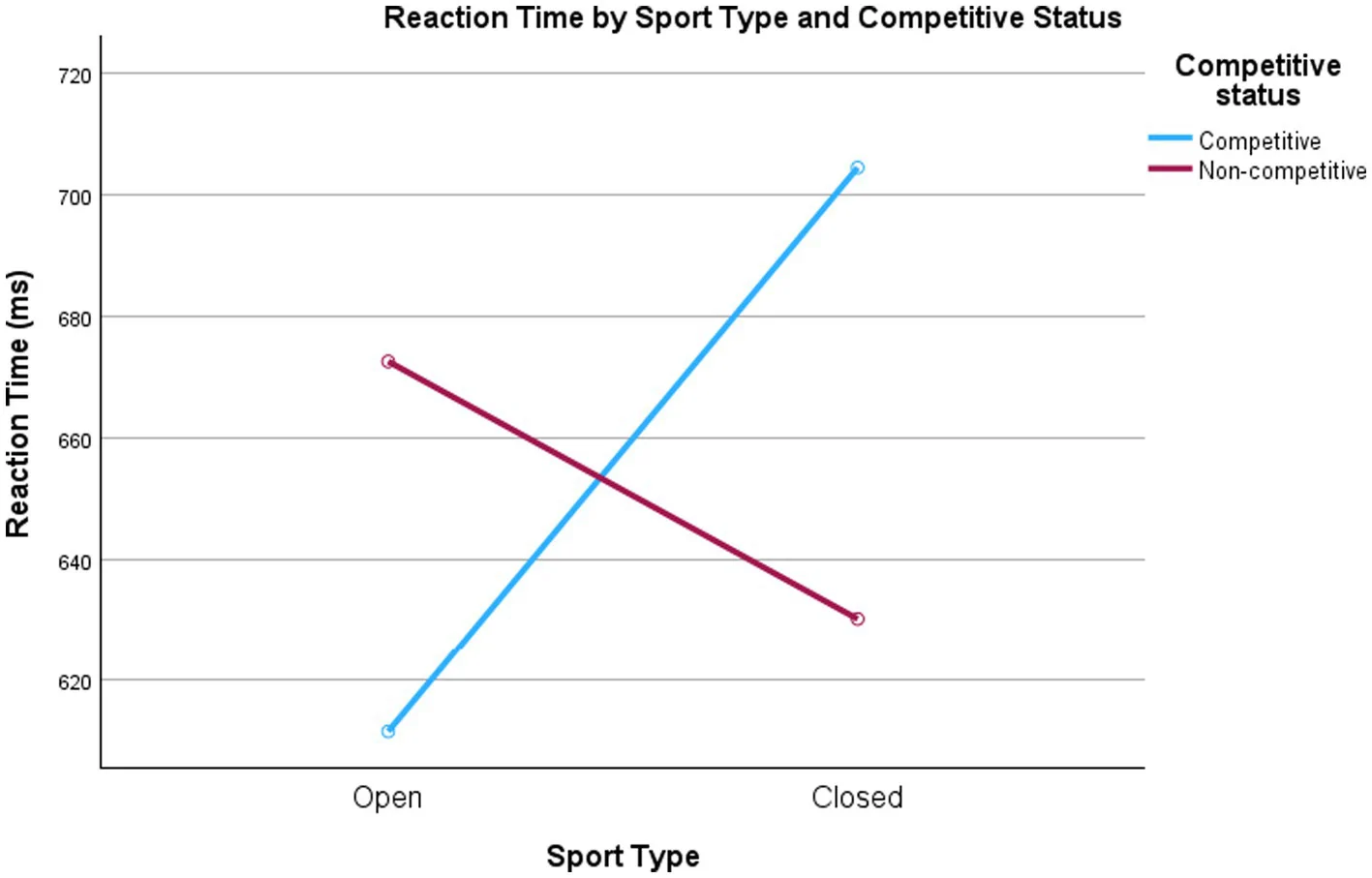
Reaction time by sport type and competitive status.

Descriptive statistics suggested a crossover pattern, whereby competitive participants demonstrated faster reaction times in open-skill sports, whereas non-competitive participants performed better in closed-skill sports (Figure 2). There was no significant main effect of sport type, *F*₍₁,₂₆₎ = 0.52, *p* = 0.477, η_p_^2^ = 0.020, nor of competitive status, *F*₍₁,₂₆₎ = 0.04, *p* = 0.854, η_p_^2^ = 0.001. The interaction between sport type and competitive status was not statistically significant, *F*₍₁,₂₆₎ = 3.71, *p* = 0.065, η_p_^2^ = 0.125.

**Figure 3:**
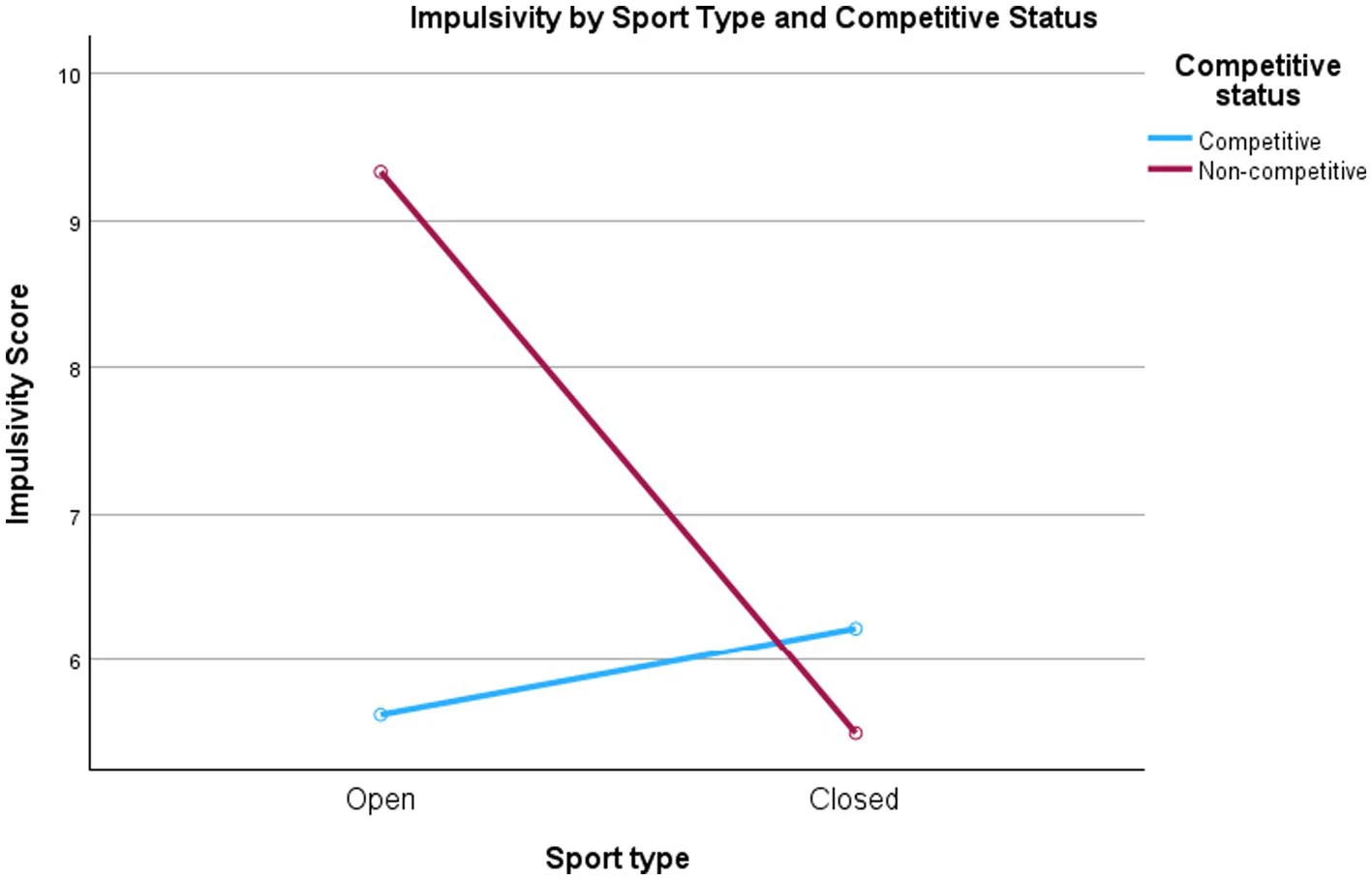
Impulsivity score by sport type and competitive status.

Non-competitive participants exhibited higher impulsivity scores in open-skill sports, whereas competitive participants showed slightly higher impulsivity in closed-skill sports (Figure 3). However, there was no significant main effect of sport type, *F*₍₁,₂₆₎ = 0.90, *p* = 0.351, η_p_^2^ = 0.034, nor of competitive status, *F*₍₁,₂₆₎ = 0.77, *p* = 0.389, η_p_^2^ = 0.029. The interaction between sport type and competitive status was also not significant, *F*₍₁,₂₆₎ = 1.69, *p* = 0.205, η_p_^2^ = 0.061.

## 4 Discussion

The study examined whether sport type (open vs. closed skill) and competitive status (competitive vs. non-competitive) influenced interference control (Stroop IF), reaction time, and impulsivity. No statistically significant main effects of either sport type or competitive status were found for any outcome variable. Open-skill athletes did not demonstrate significantly better cognitive performance than closed-skill athletes, and competitive participants did not outperform non-competitive individuals in interference control, reaction time, or impulsivity.

Another important consideration is the substantial differences in age and sex distribution between groups. Participants in the competitive and open-skill groups were, on average, older than those in the non-competitive and closed-skill groups. Moreover, the proportion of males and females differed considerably across the study groups.

Age and sex were not controlled for in the statistical analyses. Previous research suggests that both variables may influence reaction time, inhibitory control, and other executive functions in physically active individuals. Although most participants in the present study fell within an age range typically associated with peak cognitive functioning, previous research suggests that age-related variation in cognitive performance may nevertheless be present within adult populations (Tønnessen et al., 2013; Yongtawee and Woo, 2017; Tucker-Drob, 2009). Consequently, the age differences observed between groups may have influenced the cognitive outcomes and should be considered a potential confounding factor. At the same time, it is important to note that all participants were young adults, with a mean age falling within a developmental period typically associated with relatively high cognitive functioning. Therefore, age-related effects may have been less pronounced than those reported in studies involving broader age ranges.

Accordingly, the findings should be interpreted with caution. Future research should employ larger, more balanced samples, use age- and sex-matched groups, or statistically control for these variables to better isolate the effects of sport type and competitive involvement.

Despite the absence of statistically significant effects, descriptive differences in group means were observed. Participants engaged in open-skill sports showed lower mean interference scores than those in closed-skill sports, and competitive participants showed lower mean interference scores than non-competitive participants. However, none of these differences reached statistical significance.

The absence of statistically significant differences in the present study contrasts with several previous reports suggesting superior executive functioning among open-skill and competitive athletes. Several factors may explain these discrepancies. First, the sample included both competitive and non-competitive participants, representing a range of sports disciplines and performance levels, potentially increasing within-group variability and reducing the likelihood of detecting group differences. Second, the relatively small and unequal group sizes may have limited statistical power, particularly for detecting interaction effects. Although the interaction effect for reaction time yielded the largest observed effect size (η_p_^2^ = 0.125), it did not reach statistical significance. Third, methodological differences between studies should be considered. Many previous investigations have compared elite athletes with non-athletes or sedentary controls, whereas the present study compared physically active individuals differing in sport type and competitive status. As regular physical activity itself has been associated with enhanced cognitive functioning, potential cognitive advantages may have been attenuated within the current sample. Finally, differences in assessment tools, outcome measures, and task-specific cognitive demands may contribute to the inconsistent findings reported across studies.

In racket sports, which can be considered predominantly open-skill sports such as table tennis or badminton, athletes have demonstrated superior action inhibition compared to non-athletes, with higher levels of competitive participation associated with more effective response inhibition. This enhancement is observed in both domain-general and domain-specific cognitive inhibition tasks (Huang et al., 2024; Liao et al., 2017). Similarly, football players, also considered as open-skill athletes, particularly those in defensive roles, have demonstrated advanced interference control, enabling them to execute motor responses efficiently despite distracting stimuli (Wylie et al., 2018). Yao et al. (2024) examined working memory and action inhibition in Olympic athletes participating in closed-skill team sports, such as rowing and synchronized swimming. Although no significant differences in behavioral performance were observed compared to control participants, the athlete group demonstrated distinct patterns of neural activation, suggesting the use of cognitive strategies adapted to the predictable, stable demands of their sporting environment. These findings contribute to a deeper understanding of cognitive and neural adaptations in elite athletes and highlight the domain-specific nature of such enhancements (Nuri et al., 2013).

Descriptive group means for reaction time showed a crossover pattern, with competitive participants in open-skill sports exhibiting lower mean reaction times than competitive participants in closed-skill sports, whereas non-competitive participants in closed-skill sports exhibited lower mean reaction times than non-competitive participants in open-skill sports. However, the interaction between sport type and competitive status was not statistically significant. This relationship is also evident in Wang et al. (2013), where tennis players exhibited shorter reaction times than both swimmers and non-athletes. The observed advantages in open-skill athletes were evident even in a non-sport-specific task, supporting the notion that cognitive benefits derived from such training can generalize beyond the sport context. Studies suggest that athletes engaged in open-skill sports demonstrate significantly faster reaction times compared to non-athletes (Atan and Akyol, 2014; Jiang et al., 2026). Furthermore, these athletes exhibit superior performance in both the congruent and incongruent conditions of the Stroop task, as evidenced by significantly shorter reaction times than non-athletic participants (Jiang et al., 2026).

Regarding impulsivity, no statistically significant differences were observed in our study. Descriptive differences in mean impulsivity scores were observed across groups, with non-competitive participants in open-skill sports showing higher mean impulsivity scores than their counterparts, whereas competitive participants in closed-skill sports showed slightly higher mean impulsivity scores than competitive participants in open-skill sports. However, these differences were not statistically significant and should be interpreted cautiously. Svebak and Kerr (1989) support the idea that impulsive individuals tend to prefer fast-paced, high-adrenaline sports that focus on excitement and spontaneity. There is also a relationship between an athlete’s tendency to engage in careful thinking versus impulsive action and their level of athletic expertise. Vaughan et al. (2021) indicate that participation in competitive sports promotes more efficient decision-making by fostering reflective thinking while still maintaining accuracy, which is often reduced in highly impulsive decision-making. Higher levels of negative urgency (NU) and sensation seeking (SS), two subdimensions within the broader construct of impulsivity, were associated with a greater number of errors in performance. Female athletes showed higher NU than males, and lower-division athletes showed higher SS compared to top-division athletes (Millán-Sánchez et al., 2023). Overall, the findings indicate that players with higher impulsivity tend to make more mistakes in key actions. The results indicate a negative relationship between impulsivity and performance, with higher impulsivity associated with lower performance (Terres-Barcala et al., 2022). Mindfulness, including mindfulness-based training, may help counteract the negative effects of impulsivity. It appears to reduce impulsive behavior by enhancing self-reflection and improving coping effectiveness (Liang et al., 2024).

In summary, the present study did not find statistically significant effects of sport type or competitive status on cognitive performance or impulsivity. Although descriptive differences in group means were observed for some outcomes, no significant main or interaction effects were identified. Given the small and unequal group sizes, particularly in the Open-Skill Non-Competitive group, these descriptive differences should be interpreted with caution. The findings highlight the complexity of investigating cognitive performance in relation to sport participation and underscore the need for larger and more balanced samples in future research.

### 4.1 Limitations

Several limitations should be considered when interpreting the present findings. The overall sample size was relatively small (*N* = 30), which may have reduced statistical power and increased the likelihood of Type II errors. This issue is particularly relevant when examining interaction effects, which generally require larger samples to be detected reliably.

The study design also involved unequal group sizes across the four cells of the factorial design. Of particular concern was the Open-Skill Non-Competitive group, which included only three participants. Such a small cell size may reduce the stability of group estimates and interaction effects and may disproportionately influence the observed data patterns. Although descriptive reaction time data showed a crossover pattern across groups, no statistically significant interaction effect was identified. These findings should be interpreted cautiously, as estimates derived from small and unequal groups are more susceptible to sampling variability and the influence of individual cases.

Differences in age and sex distribution across groups represent an additional limitation. Competitive participants were generally older than non-competitive participants, and the proportion of males and females varied substantially between groups. Because age and sex were not statistically controlled, these variables may have acted as confounding factors. Consequently, some of the observed differences in reaction time, inhibitory control, and impulsivity may partly reflect age- or sex-related influences rather than the effects of sport type or competitive status alone.

Taken together, the small and unequal group sizes, along with the demographic differences between groups, limit the robustness of the estimated interaction effects and reduce the generalizability of the findings. Future research should employ larger and more balanced samples, use age- and sex-matched groups, and statistically control for potential confounding variables in order to more clearly isolate the effects of sport type and competitive involvement on cognitive performance.

## 5 Conclusion

The present study found no evidence that sport type or competitive status influences interference control, reaction time, or impulsivity. These findings highlight the complexity of the relationship between sport participation and cognitive performance. Given the limited and uneven sample distribution, future studies using larger, more balanced samples and more homogeneous sport groups are needed to clarify the potential role of sport participation characteristics in cognitive functioning and impulsivity.

## Data Availability

The raw data supporting the conclusions of this article will be made available by the authors, without undue reservation.

## References

[ref1] Atan T. Akyol P. (2014). Reaction times of different branch athletes and correlation between reaction time parameters. Procedia - social and behavioral sciences in 5th World Conference on Educational Sciences, (Amsterdam, The Netherlands: Elsevier B.V.), 2886–2889.

[ref2] Hagyard J. Brimmell J. Edwards E. J. Vaughan R. S. (2021). Inhibitory control across athletic expertise and its relationship with sport performance. J. Sport Exerc. Psychol. 43, 14–27. doi: 10.1123/jsep.2020-0043, 33383568

[ref3] Heilmann F. Weinberg H. Wollny R. (2022). The impact of practicing open- vs. closed-skill sports on executive functions—a Meta-analytic and systematic review with a focus on characteristics of sports. Brain Sci. 12:1071. doi: 10.3390/brainsci12081071, 36009134 PMC9406193

[ref4] Holfelder B. Klotzbier T. J. Eisele M. Schott N. (2020). Hot and cool executive function in elite- and amateur- adolescent athletes from open and closed skills sports. Front. Psychol. 11:694. doi: 10.3389/fpsyg.2020.00694, 32373029 PMC7177013

[ref5] Huang Q. Mao X. Shi J. Pan J. Li A. (2024). Enhanced cognitive inhibition in table tennis athletes: insights from color-word and spatial Stroop tasks. Brain Sci. 14:443. doi: 10.3390/brainsci14050443, 38790422 PMC11117886

[ref6] Jiang Y. Wu Y. Zou F. Li W. (2026). Impact of open and closed-skill sports on inhibitory control: evidence from event related potentials. Int. J. Sport Exerc. Psychol. 24, 620–639. doi: 10.1080/1612197X.2025.2477162

[ref7] Liang P. Jiang H. Wang H. Tang J. (2024). Mindfulness and impulsive behavior: exploring the mediating roles of self-reflection and coping effectiveness among high-level athletes in Central China. Front. Psychol. 15:1304901. doi: 10.3389/fpsyg.2024.1304901, 38283206 PMC10808761

[ref8] Liao K.-F. Meng F.-W. Chen Y.-L. (2017). The relationship between action inhibition and athletic performance in elite badminton players and non-athletes. J. Hum. Sport Exerc. 12, 574–581. doi: 10.14198/jhse.2017.123.02

[ref9] Ludyga S. Gerber M. Pühse U. Looser V. N. Kamijo K. (2020). Systematic review and meta-analysis investigating moderators of long-term effects of exercise on cognition in healthy individuals. Nat. Hum. Behav. 4, 603–612. doi: 10.1038/s41562-020-0851-8, 32231280

[ref10] Mandolesi L. Polverino A. Montuori S. Foti F. Ferraioli G. Sorrentino P. . (2018). Effects of physical exercise on cognitive functioning and wellbeing: biological and psychological benefits. Front. Psychol. 9:509. doi: 10.3389/fpsyg.2018.00509, 29755380 PMC5934999

[ref11] McKinney J. Velghe J. Fee J. Isserow S. Drezner J. A. (2019). Defining athletes and exercisers. Am. J. Cardiol. 123, 532–535. doi: 10.1016/j.amjcard.2018.11.001, 30503799

[ref12] Millán-Sánchez A. Madinabeitia I. de la Vega R. Cárdenas D. Ureña A. (2023). Effects of emotional regulation and impulsivity on sports performance: the mediating role of gender and competition level. Front. Psychol. 14:1164956. doi: 10.3389/fpsyg.2023.1164956, 37469888 PMC10352320

[ref13] Nuri L. Shadmehr A. Ghotbi N. Attarbashi Moghadam B. (2013). Reaction time and anticipatory skill of athletes in open and closed skill-dominated sport. Eur. J. Sport Sci. 13, 431–436. doi: 10.1080/17461391.2012.738712, 24050458

[ref14] Pačesová P. (2021). Cognitive and executive functions of young men regarding sport activity and personality traits. Sustainability 13:11752. doi: 10.3390/su132111752

[ref15] Ren S. Shi P. Feng X. Zhang K. Wang W. (2024). Executive function strengths in athletes: a systematic review and meta-analysis. Brain Behav. 15:e70212. doi: 10.1002/brb3.70212, 39740775 PMC11688047

[ref16] Scharfen H.-E. Memmert D. (2019). Measurement of cognitive functions in experts and elite athletes: a meta-analytic review. Appl. Cogn. Psychol. 33, 843–860. doi: 10.1002/acp.3526

[ref17] Svebak S. Kerr J. (1989). The role of impulsivity in preference for sports. Personal. Individ. Differ. 10, 51–58. doi: 10.1016/0191-8869(89)90177-3, 42475469

[ref18] Terres-Barcala L. Albaladejo-Blázquez N. Aparicio-Ugarriza R. Ruiz-Robledillo N. Zaragoza-Martí A. Ferrer-Cascales R. (2022). Effects of impulsivity on competitive anxiety in female athletes: the mediating role of mindfulness trait. Int. J. Environ. Res. Public Health 19:3223. doi: 10.3390/ijerph19063223, 35328913 PMC8951821

[ref19] Tønnessen E. Haugen T. Shalfawi S. (2013). Reaction time aspects of elite sprinters in athletics world championships. J. Strength Cond. Res. 27, 885–892. doi: 10.1519/JSC.0b013e31826520c3, 22739331

[ref20] Tucker-Drob E. M. (2009). Differentiation of cognitive abilities across the life span. Dev. Psychol. 45, 1097–1118. doi: 10.1037/a0015864, 19586182 PMC2855504

[ref21] Vaughan R. S. Hagyard J. D. Edwards E. J. Jackson R. C. (2021). Reflection-impulsivity in athletes: a cross-sectional and longitudinal investigation. Eur. J. Sport Sci. 21, 1436–1447. doi: 10.1080/17461391.2020.1861106, 33284734

[ref22] Wang C.-H. Chang C.-C. Liang Y.-M. Shih C.-M. Chiu W.-S. Tseng P. . (2013). Open vs. closed skill sports and the modulation of inhibitory control. PLoS One 8:e55773. doi: 10.1371/journal.pone.0055773, 23418458 PMC3572130

[ref23] Wylie S. A. Bashore T. R. Van Wouwe N. C. Mason E. J. John K. D. Neimat J. S. . (2018). Exposing an “intangible” cognitive skill among collegiate football players: enhanced interference control. Front. Psychol. 9:49. doi: 10.3389/fpsyg.2018.00049, 29479325 PMC5811505

[ref24] Yao Z.-F. Sligte I. G. Ridderinkhof R. (2024). Olympic team rowers and team swimmers show altered functional brain activation during working memory and action inhibition. Neuropsychologia 203:108974. doi: 10.1016/j.neuropsychologia.2024.108974, 39182905

[ref25] Yongtawee A. Woo M.-J. (2017). The influence of gender, sports type and training experience on cognitive functions in adolescent athletes. Exerc. Sci. 26, 159–167. doi: 10.15857/ksep.2017.26.2.159

[ref26] Zhou Q. Chen S. H. Main A. (2012). Commonalities and differences in the research on children’s effortful control and executive function: a call for an integrated model of self-regulation. Child Dev. Perspect. 6, 112–121. doi: 10.1111/j.1750-8606.2011.00176.x

